# Blue Light-Dependent Pre-mRNA Splicing Controls Pigment Biosynthesis in the Mushroom *Terana caerulea*

**DOI:** 10.1128/spectrum.01065-22

**Published:** 2022-09-12

**Authors:** Stefanie Lawrinowitz, Jacob M. Wurlitzer, Dieter Weiss, Hans-Dieter Arndt, Erika Kothe, Markus Gressler, Dirk Hoffmeister

**Affiliations:** a Friedrich-Schiller-Universität Jena, Institute of Pharmacy, Jena, Germany; b Pharmaceutical Microbiology, Leibniz Institute for Natural Product Research and Infection Biology, Hans Knöll Institute, Jena, Germany; c Friedrich-Schiller-Universität Jena, Institute for Organic Chemistry and Macromolecular Chemistry, Jena, Germany; d Friedrich-Schiller-Universität Jena, Institute for Microbiology, Jena, Germany; University of Molise

**Keywords:** Basidiomycota, corticin, light, natural products, splicing

## Abstract

Light induces the production of ink-blue pentacyclic natural products, the corticin pigments, in the cobalt crust mushroom Terana caerulea. Here, we describe the genetic locus for corticin biosynthesis and provide evidence for a light-dependent dual transcriptional/cotranscriptional regulatory mechanism. Light selectively induces the expression of the *corA* gene encoding the gateway enzyme, the first described mushroom polyporic acid synthetase CorA, while other biosynthetic genes for modifying enzymes necessary to complete corticin assembly are induced only at lower levels. The strongest *corA* induction was observed following exposure to blue and UV light. A second layer of regulation is provided by the light-dependent splicing of the three introns in the pre-mRNA of *corA*. Our results provide insight into the fundamental organization of how mushrooms regulate natural product biosynthesis.

**IMPORTANCE** The regulation of natural product biosyntheses in mushrooms in response to environmental cues is poorly understood. We addressed this knowledge gap and chose the cobalt crust mushroom *Terana caerulea* as our model. Our work discovered a dual-level regulatory mechanism that connects light as an abiotic stimulus with a physiological response, i.e., the production of dark-blue pigments. Exposure to blue light elicits strongly increased transcription of the gene encoding the gateway enzyme, the polyporic acid synthetase CorA, that catalyzes the formation of the pigment core structure. Additionally, light is a prerequisite for the full splicing of *corA* pre-mRNA and, thus, its proper maturation. Dual transcriptional/cotranscriptional light-dependent control of fungal natural product biosynthesis has previously been unknown. As it allows the tight control of a key metabolic step, it may be a much more prevalent mechanism among these organisms.

## INTRODUCTION

Light is an abiotic environmental factor of utmost importance. Beyond the phototrophic lifestyle that uses the energy of visible light for reducing carbon dioxide during photosynthesis, light determines the metabolism of photo- and heterotrophic organisms, for example, during morphogenesis or the circadian rhythm (1). These functions necessitate the light-controlled regulation of gene expression. Beyond plants, animals, and humans, light also greatly affects fungi and is known to cue physiological responses (2). For example, the production of the aflatoxin-type metabolite sterigmatocystin in the filamentous fungus Aspergillus nidulans was found to be decreased when the fungus was exposed to white light (3). Such genetically well-understood phenotypes generally pertain to the ascomycetes, i.e., one of the two large groups of higher fungi.

With more than 30,000 species, the basidiomycetes represent the second group. They are prolific producers of bioactive and highly functionalized natural products as well (4). Light perception in basidiomycetes has traditionally been studied in the context of morphogenesis, where blue and UV light were found to induce carpophore formation in Schizophyllum commune (5). This fungus was also instrumental in identifying the basidiomycete blue-light receptor, a white collar 1/2 (WC-1/2)-like system for the transcriptional control of the target genes (6). However, only limited studies are available on basidiomycete gene regulation, in particular regarding natural products and light-dependent control mechanisms.

**FIG 1:**
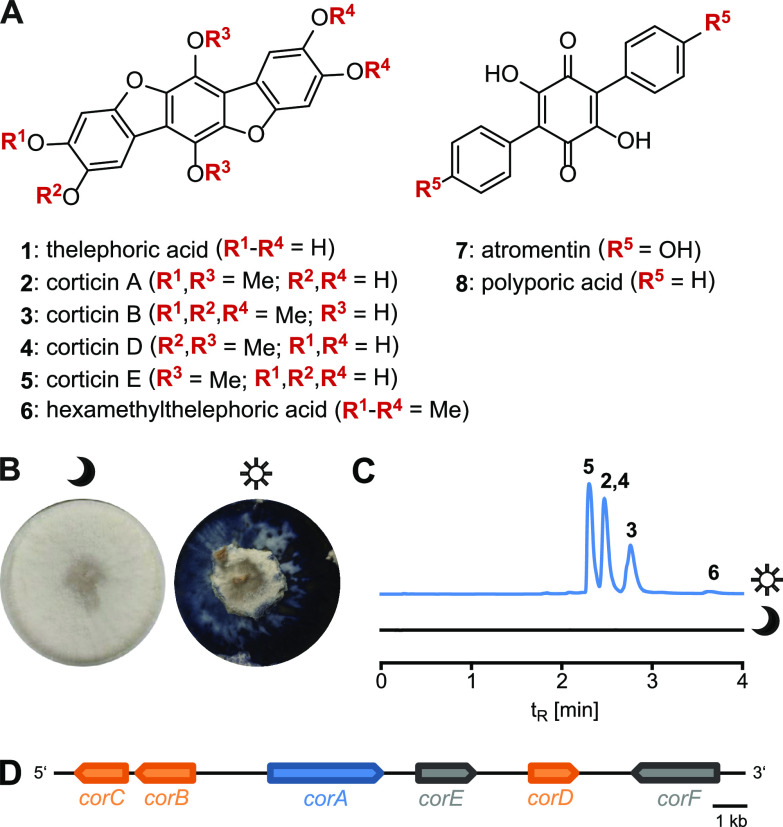
Terphenylquinone mushroom pigments. (A) Chemical structures of mushroom terphenylquinones and their biosynthesis intermediates. (B) *Terana caerulea* mycelium kept continuously in the dark for 21 days (colorless) (left) or kept in the dark for 14 days, followed by light exposure for 7 days (ink-blue pigmentation) (right). (C) Chromatographic analysis of corticins 2 to 6, extracted from a *T. caerulea* mycelium grown under dark conditions (black) (bottom chromatogram) or light conditions (blue) (top chromatogram). Chromatograms were recorded at a λ of 358 nm. (D) The 20-kb genetic locus for corticin biosynthesis in *Terana caerulea*. Arrows indicate the transcriptional direction of the genes. For clarity, introns are not shown. Blue, the gene for polyporic acid synthetase; orange, putative *O*-methyltransferase genes; gray, genes of unknown function, tentatively assigned to corticin biosynthesis.

To gain insight into the light-dependent regulation of mushroom metabolism, the cobalt crust mushroom Terana caerulea was chosen as a significant model. It proliferates on decaying hardwood, with a worldwide distribution at low altitudes in mild climates. Its common name refers to its most remarkable physiological feature, which is the phenomenally dark-blue pigmentation of its crust-like fruiting bodies. Its ink-blue pigments, the thelephoric acid (compound 1)-derived corticins (compounds 2 to 6) (Fig. 1A) (7–9), appear only when the mycelium is exposed to light, while it remains white in the dark (10) (Fig. 1B). Based on previous work, reviewed by Gill and Steglich (11), corticin biosynthesis is expected to proceed through a *para*-terphenylquinone intermediate, most likely atromentin (compound 7). Extending from available genetic and biochemical data for terphenylquinone biosynthesis in other fungi (12–15), and given the light-dependent pigmentation, T. caerulea seems a plausible choice to study the light-dependent regulation of biosynthesis genes in basidiomycetes. Here, we report that the transcription of the *corA* gene, encoding the terphenylquinone synthetase as a gateway enzyme for corticin biosynthesis that produced polyporic acid (compound 8) is induced by blue or UV light. Simultaneously, the full splicing of *corA* pre-mRNA also depends on exposure to light. This unprecedented light-dependent dual transcriptional/cotranscriptional regulatory mechanism of a fungal gene allows the tight control of a key physiological step and, hence, may represent a more widespread genetic control mechanism in basidiomycetes.

## RESULTS

### Identification of the corticin biosynthesis gene cluster.

First, we confirmed that the strain used in our study, *T. caerulea* CBS 452.86, produced corticins in a light-dependent manner (Fig. 1B and C; see also Table S1 in the supplemental material). To investigate the genetic basis of corticin biosynthesis, the genome of this fungus was sequenced. The sequence data showed a 53.6% GC content, and the sequence was assembled into 1,226 contigs covering 50.86 Mb (GenBank accession number JAKOGH000000000). Bioinformatic analysis revealed a genetic locus (Fig. 1D) encoding enzymes with a plausible function in corticin biosynthesis. As the chemical structure of the corticins (Fig. 1A) derives from a terphenylquinone, a genetic locus that encodes a terphenylquinone synthetase was expected. These enzymes follow a characteristic tridomain architecture (12–15) that features adenylation, thiolation, and thioesterase domains. Their genes are therefore easily recognizable during bioinformatic screening of genomic sequence data. A single gene with homology to a tridomain terphenylquinone synthetase gene, now referred to as *corA*, was present in the *T. caerulea* genome. The CorA enzyme is encoded by a 2,985-bp open reading frame (Fig. 1D) (GenBank accession number OM515349), interrupted by three predicted introns. Phylogenetic analyses of the adenylation domains of CorA and other fungal tridomain synthetases showed dichotomous evolution into an ascomycete versus a basidiomycete branch. Interestingly, CorA has a position basal to the ascomycete branch (Fig. S1). Consistent with the corticins being *O*-methylated compounds, genes for three putative *O*-methyltransferases (CorB, CorC, and CorD) were identified adjacent to *corA*. Two additional reading frames (*corE* and *corF*) in the immediate proximity lack obvious homologies. Subsequently, we intended to verify that the correct locus had been found by (i) the heterologous reconstitution of CorA activity, i.e., of the central biosynthesis enzyme, and (ii) the evaluation of the light-dependent regulation of biosynthetic genes.

### CorA is a polyporic acid synthetase.

**FIG 2:**
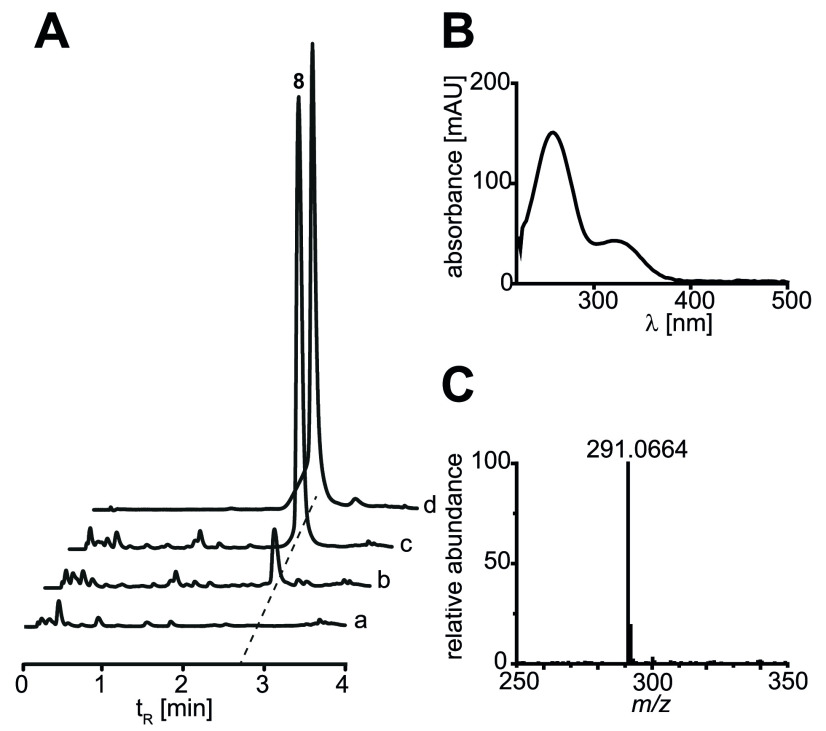
Natural product analysis: chromatographic analysis of polyporic acid (compound 8) formation *in vivo* by recombinant Aspergillus nidulans strains tStL04 and tStL06. (A) Chromatograms of crude extracts, recorded at a λ of 254 nm. Trace a, negative control (untransformed wild-type A. nidulans FGSC A4); trace b, A. nidulans tStL06 (carrying *corA* gDNA); trace c, A. nidulans tStL04 (*corA* cDNA); trace d, authentic compound 8. (B) UV-visible (UV-Vis) spectrum of the new UHPLC signal (*t_R_* = 2.8 min) in the tStL04 extract. (C) High-resolution MS spectrum of the signal at a *t_R_* of 2.8 min.

To clarify its function, the CorA protein was produced by heterologous overexpression in its native form in Aspergillus nidulans tStL04 and tStL06. These expression strains harbored a cDNA or a chromosomal copy of *corA*, respectively, integrated into the genome under the control of the alcohol-inducible promoter P*alcA* (16, 17). Heterologously produced CorA led to a chromatographically detectable compound at a retention time (*t_R_*) of 2.8 min (Fig. 2A) in A. nidulans extracts of both strains, while it was absent in extracts of the wild-type control strain (A. nidulans FGSC A4). The respective compound was isolated from ethyl acetate extracts of the culture broth of A. nidulans tStL04 and identified as compound 8 by using ultrahigh-performance liquid chromatography–mass spectrometry (UHPLC-MS), liquid chromatography-tandem mass spectrometry (LC-MS/MS), and nuclear magnetic resonance (NMR) spectroscopy (Fig. 2; Fig. S2 and Table S2). Moreover, the experimental data for compound 8 were in full agreement with previously reported values for polyporic acid (18, 19), and we confirmed this identity with an authentic sample. Notably, this finding was in contrast to the previous hypothesis that corticin biosynthesis starts from l-tyrosine, proceeding via 4-hydroxyphenylpyruvate and atromentin (compound 7) as intermediates (11). To test if corticins originate from l-phenylalanine and are formed from compound 8 (Fig. 1A) via phenylpyruvate as an intermediate in the native producer, *T. caerulea* was cultivated on agar plates supplemented with l-[3-^13^C]phenylalanine. Mass spectrometry analysis of ethyl acetate extracts confirmed a mass shift of corticin pigments 2 and 4 to *m/z* 396 and 397 (unlabeled, *m/z* 395), strongly indicating the incorporation of one or two ^13^C atoms from one or two labeled precursors (Fig. S3) during biosynthesis. The incorporation ratio for *m/z* 396 and 397 were 62.9 and 86.2%. Furthermore, in a parallel stable-isotope labeling study, *T. caerulea* cultures were alternatively supplemented with l-[3,5-D_2_]tyrosine. In this experiment, a mass shift was not observed, but we noticed that corticin production was significantly reduced (Fig. S3).

### A PCR experiment suggests two dissimilar mechanisms for the light-dependent regulation of *corA* expression.

**FIG 3:**
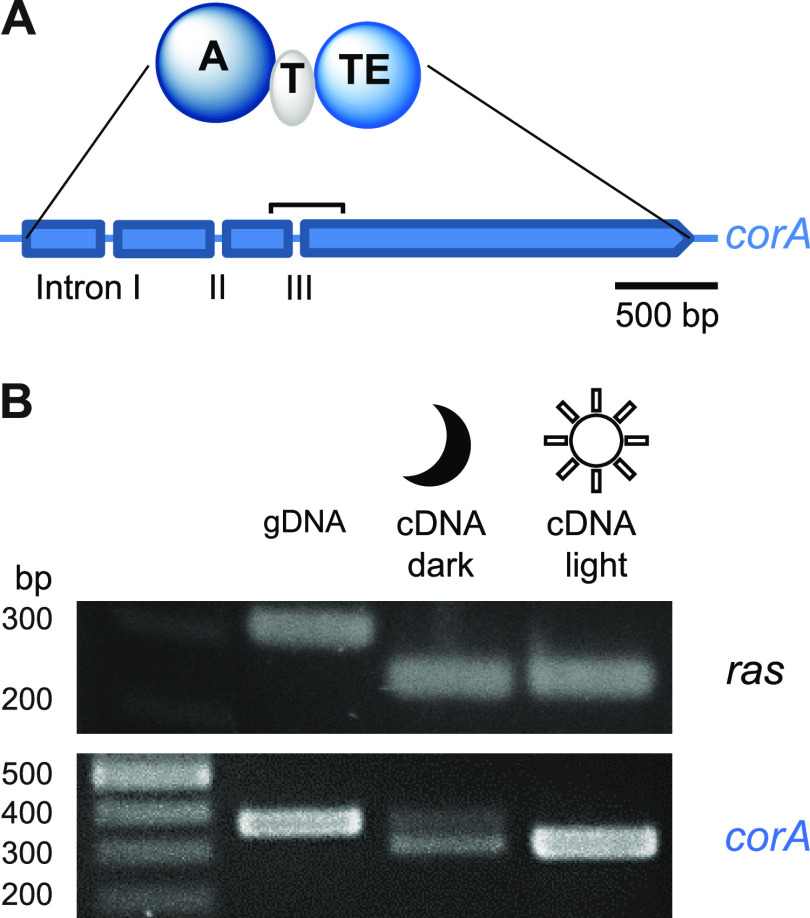
The polyporic acid synthetase CorA. (A) Tridomain structure of the enzyme. The intron-exon pattern of the *corA* gene is shown below. Intron positions are shown as spaces between arrow segments. (B) Expression of *corA* under light and dark conditions, as assessed by semiquantitative reverse transcription-PCR, whose amplicon (marked by the horizontal bracket in panel A) spans intron III, and subsequent gel electrophoretic analysis. The amplicon length for *corA* is 368 or 308 bp. As a control, the constitutively expressed *ras* gene was included, whose amplicons are 312 or 255 bp long. A, adenylation; T, thiolation; TE, thioesterase/cyclase.

Given the light-dependent blueing of *T. caerulea*, the gene locus was expected to show light-regulated expression of the *cor* genes. To gain first insights, semiquantitative reverse transcription-PCR was performed on *corA* from mycelia grown in the presence or absence of white light. This PCR covered intron III, i.e., the intron located toward the 3′ end of *corA* (Fig. 3A). Consistent with our assumption, *corA* gene expression was increased in mycelia that were exposed to light, compared to the control grown in the dark (Fig. S4). For all other *cor* genes, semiquantitative reverse transcription-PCR indicated minimally increased or unchanged expression levels. Interestingly, we noticed by semiquantitative PCR that the expression pattern depended on light as well. A single amplicon (308 bp) was detected when the light-exposed mycelium was used as the origin of the PCR template, whereas two amplicons of different lengths (308 and 368 bp) were detected when the mycelium had been grown in the dark (Fig. 3B). Sequencing of the two PCR fragments verified that the difference correlated with the loss or retention of intron III. This finding suggested the two-layered light-dependent control of *corA* expression. Beyond the primary transcriptional and quantitative effects that lead to an increased amount of the *corA* transcript, light apparently impacted splicing, a qualitative co- or posttranscriptional secondary effect.

### Expression of the gateway corticin biosynthesis gene *corA* depends on blue light.

**FIG 4:**
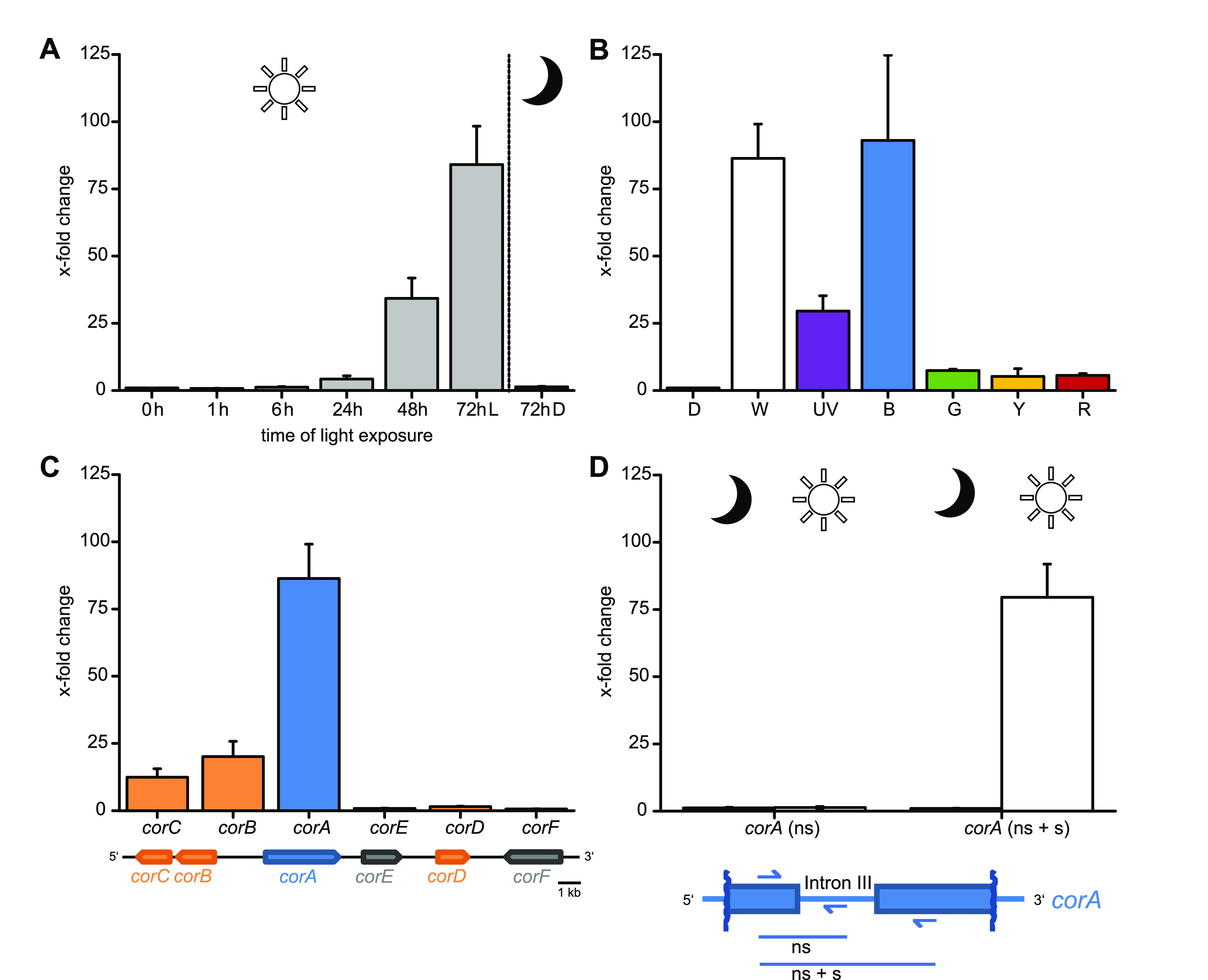
Quantitative analysis of *cor* gene expression by qRT-PCR. (A) Time-dependent gene expression of *corA* after growth with white-light/dark cycles. (B) Relative transcript levels of *corA* after exposure to different wavelengths. D, dark; W, white light; B, blue (λ = 465 nm); G, green (λ = 525 nm); Y, yellow (λ = 590 nm); R, red (λ = 625 nm). The wavelength for UV light is 367 nm. (C) Expression of *cor* genes after 72 h of exposure to white-light/dark cycles. (D) Quantitative assessment of *corA* transcript splicing after 72 h of dark and light conditions using intron III as a readout. A mycelium kept in darkness served as a negative control. Oligonucleotides binding in the exon portion are oSS07 and oSS72, while oSS140 binds in intron III. Values shown are mean *x*-fold changes and standard errors of the means. ns, nonspliced; s, spliced. The values are normalized to the expression of *ras* as a control gene.

We sought to clarify if *corA* expression and proper splicing are, in fact, regulated by two different light-dependent mechanisms. First, the quantitative effect of light was investigated, and quantitative real-time PCR (qRT-PCR) was performed with *T. caerulea* RNA that had been isolated from mycelium grown in an alternating 12-h white-light/dark cycle. After 24 h (48 h), the *corA* gene expression level had increased 4.2-fold (34-fold) and culminated with an 84-fold increase after 72 h, compared to the control kept in constant darkness (1.4-fold increase after 72 h) (Fig. 4A). Short-time continuous light (1 h and 6 h, respectively) did not alter *corA* gene expression (0.8-fold and 1.2-fold).

Additionally, the wavelength (λ) that induced transcription was investigated. *T. caerulea* was exposed to monochromatic light, and we chose cycles of 8 h of light/16 h of darkness, for 72 h. To prevent the detrimental effects of mutagenesis, a 1-h light/23-h darkness interval was applied for UV light. The *corA* gene was strongly induced by UV (λ = 367 nm) and blue (λ = 465 nm) light, with 29-fold and 93-fold increases in the transcript levels, respectively. However, green (λ = 525 nm), yellow (λ = 590 nm), and red (λ = 625 nm) light led to only minor effects (7.5-fold, 5.3-fold, and 5.7-fold, respectively) (Fig. 4B). In summary, these data convincingly show that the *corA* transcript level is a function of exposure to blue and UV light.

Subsequently, we investigated if light impacted the expression of *corB* and *corC*, encoding putative *O*-methyltransferases. In comparison to *corA*, these genes were less strongly yet still distinctly upregulated under white light after 72 h (20-fold and 12-fold, respectively) (Fig. 4C), while the transcription of the adjacent reading frames (*corD*, *corE*, and *corF*) did not respond to light exposure at all. These data confirmed that light controls corticin biosynthesis primarily by the transcriptional control of *corA*, i.e., the gene encoding the gateway enzymatic step, and that prolonged light exposure is necessary for fully induced expression. Notably, homologs of the genes coding for white collar-like fungal blue-light receptor proteins (WC-1 and WC-2) (20) were identified in the genome of *T. caerulea* as well (Table S3 and Fig. S5). A bioinformatically predicted *wc-1* homolog (*twc1*) encodes a putative sensory protein (1,086 amino acids [aa]) that features three PAS folds (named after the *Drosophila* proteins period (PER), aryl hydrocarbon receptor nuclear translocator [ARNT], and single-minded protein [SIM]) that promote protein-protein interactions. One of the predicted PAS folds belongs to a specialized subgroup, the LOV domain (light, oxygen, and voltage sensing), i.e., portions of the light receptor that were shown to sense blue light (maximum absorption at 465 nm) by a flavin cofactor (20, 21). The putative *wc-2* homolog (*twc2*) encodes a 412-aa protein containing one PAS fold and one C-terminal GATA-type zinc finger. Both predicted proteins share the domain arrangement with other basidiomycete white collar-like proteins (6, 22, 23) (Table S3).

### Light is a prerequisite for complete *corA* splicing.

Next, the phenomenon of the different transcript lengths under dark and light conditions was analyzed in greater detail. Semiquantitative reverse transcription-PCR disclosed the light-dependent splicing of *corA* intron III that led to two transcript variants, the fully spliced and the intron III-retaining variants, in the dark, while only the fully spliced transcript version was observed after light exposure. To clarify if the phenomenon observed for intron III occurs with the other introns as well, we additionally investigated introns I and II. The same light-dependent splicing phenomenon was observed for both intron I and intron II of *corA* (Fig. S6), and full splicing is required to prevent the premature termination of translation due to stop codons present in the intron sequences.

Additionally, the amounts of intron-retained and spliced *corA* transcripts under dark and light conditions were quantified by qRT-PCR, choosing the presence or absence of intron III as a readout. To this end, two oligonucleotide primer pairs were used, which amplify either only intron-containing, nonspliced transcripts (annealing to exon III and intron III) (Fig. 4D) or both transcript variants with and without the remaining intron, hence differentiating them by size (annealing to exons III and IV up- and downstream of intron III). We hypothesized that the number of transcripts in which introns were retained would be negatively correlated with increasing levels of the spliced transcript under white-light conditions. Surprisingly, exposure to light strongly increased the amount of the spliced transcript (79-fold), whereas the amount of the nonspliced transcript remained virtually unchanged (Fig. 4D). This finding suggests that the fungal cells maintain low basal levels of nonspliced *corA* transcripts independent of light exposure. In summary, light is required to induce both the strong transcriptional upregulation of *corA* and complete cotranscriptional splicing leading to functional mRNA and a catalytically active CorA enzyme to affect the blue corticin-mediated pigmentation of fungal hyphae.

## DISCUSSION

Our results demonstrate the key role of the terphenylquinone synthetase CorA and its product (compound 8) in corticin biosynthesis. Previously characterized mushroom terphenylquinone synthetases share an identical set of three catalytic domains with CorA (adenylation-thiolation-thioesterase domains) (Fig. 3A) yet catalyze the biosynthesis of compound 7 (12–15). Our finding indicates that mushroom tridomain nonribosomal peptide synthetase-like enzymes are functionally more diverse than evident from their amino acid sequence or domain setup. CorA represents the first characterized polyporic acid synthetase from a basidiomycete. Yet our finding somewhat resembles the ascomycete situation where tridomain nonribosomal peptide synthetase-like enzymes were first recognized as bisindolylquinone synthetases (24–26), while later work revealed more diverse activities, among them terphenylquinone and furanone synthetase (18, 27). The finding that l-phenylalanine, but not l-tyrosine, serves as a biosynthetic source of the corticins seems counterintuitive from a biosynthetic viewpoint as it implies repeated monooxygenase activity downstream of the CorA-mediated formation of compound 8. A corresponding gene for a backbone oxidizing enzyme (or more than one gene/enzyme) is not encoded in proximity to *corA*, which may point to an auxiliary genetic locus that is also dedicated to corticin formation. In contrast, putative *S*-adenosylmethionine-dependent *O*-methyltransferase genes whose expression is weakly responsive to light are clustered with *corA*. A nonclustered, scattered arrangement of biosynthetic genes is frequently observed in basidiomycetes (4).

Our study discovered that light and the biosynthesis of a mushroom pigment are functionally connected by both transcriptional activation and correct splicing of the pre-mRNA of the gateway enzyme CorA. For green plants, such light-controlled transcriptional regulation connected to concomitant splicing regulation as well as the link of red light, absorbed by phytochromes, and alternative splicing have been described (28–31). Fungi, as nonphotoautotrophic organisms, have evolved the capacity to sense and respond to light of several wavelengths as well. In the mold and model ascomycete A. nidulans, red light is sensed by phytochromes and impacts developmental events; e.g., it favors asexual while it represses sexual propagation (32). Furthermore, a recent study revealed that red light controls the expression of about 10% of the entire gene set of this filamentous fungus (33, 34). The Neurospora crassa circadian rhythm is another important fungal example of light and correlated gene expression that has been studied extensively (35, 36). Blue (and near-UV) light is sensed by the white collar 1 (WC-1) protein, which features a flavin adenine dinucleotide chromophore and carries a C-terminal zinc finger DNA-binding domain (20). WC-1 forms a complex with WC-2 that possesses a DNA-binding domain as well and is required for stabilization and proper DNA binding that mediate the hierarchical light-dependent transcription of, e.g., other transcription factor genes, which then control the expression of target genes after translocation to the nucleus (37–39).

Both phytochrome-like red-light and WC-like blue-light receptors as well as light-dependent effects on development and metabolism were found in basidiomycetes as well (6, 40). During this study, genes orthologous to *wc-1* and *wc-2* were identified in the *T. caerulea* genome (*twc1* and *twc2*). Their products may represent the beginning of the as-yet-unknown signal transduction cascade, as blue and near-UV light, i.e., the wavelengths sensed by WC-1, induced both the transcription of the gene for polyporic acid synthetase, *corA*, and the proper splicing of its pre-mRNA.

In the plant Arabidopsis thaliana, light-dependent alternative splicing by the inclusion of an additional exon and the use of alternative splicing sites decides whether the transcript is retained in the nucleus, degraded, or, in the case of the removal of the exon, spliced to the coding isoform and translocated to the cytoplasm for translation. Furthermore, the involvement of the target of rapamycin signaling pathway was shown previously (30). Signal transduction downstream of the photoreceptor to the target genes is generally little understood in basidiomycetes, yet evidence exists that light controls their metabolism (41). In the oyster mushroom Pleurotus ostreatus, the exposure of mycelia to blue light led to the massive accumulation of shikimic acid, a central intermediate of aromatic amino acid biosynthesis (42). For ascomycetes, A. nidulans with its velvet complex VelB/VeA and LaeA, the global regulator of secondary metabolism, was pivotal in unraveling the triple interconnection of light with the development and regulation of natural product metabolism (43). A comparative transcriptomic study confirmed the role of light in the regulation of carotene and other natural product genes by comparing wild-type Fusarium fujikuroi to a *wcoA* mutant that lost the functional white collar 1 protein (44).

Interestingly, continuous exposure to light of <6 h did not induce *corA* expression. This slow transcriptional response is in contrast to observations for, e.g., Aspergillus fumigatus, whose light regulator gene *lreA* is induced after 15 min of exposure to light, reaches its maximum after 60 min, and nearly remains there for another hour (45). In N. crassa, a light-dependent transcriptional response is seen within 15 min or less, and approximately 400 target genes of the white collar complex were identified after 15 min of illumination (46). In the case of *corA*, the required long-term exposure (>12 h) to light may point to either the slow formation of the blue-light receptor complex or, mediated by an unknown cascade, a low affinity of the respective transcription factor for its promoter. The corticins, the products of light-induced metabolism, are stable, long-lived molecules that are not rapidly turned over, as evident by the constant ink-blue mycelia. The slow induction of *corA* may represent a genetic mechanism to prevent pigmentation after short-term light exposure that helps reduce an unnecessary metabolic burden.

Fungal secondary metabolism is controlled by pathway-specific and global transcriptional regulators (47–49). A further layer of regulation includes chromatin-mediated epigenetic modification, which has been shown, e.g., for the biosynthesis of sterigmatocystin and several other compounds (3, 50, 51). Also, RNA editing, as a posttranscriptional regulatory mechanism, was reported to impact triterpene metabolism in the basidiomycete mushroom Ganoderma lucidum (52) yet mostly in the context of ascomycete development (53). Alternative splicing controls fungal metabolism as well. In the context of catabolism, the lignocellulose-degrading mushroom Phanerochaete chrysosporium showed substrate-dependent alternative splicing of a gene encoding exocellobiohydrolase, which hypothetically changes the enzyme’s substrate preferences (54). Alternative splicing has also been recognized as an important regulatory mechanism in the human-pathogenic basidiomycetous yeast Cryptococcus neoformans (55) and is well established in numerous ascomycetes, among them Sclerotinia sclerotiorum (56), Sordaria macrospora (57), and aspergilli (58, 59). However, these and other reports on alternative splicing pertain to developmental or metabolic aspects, but none of them pertain to the regulation of natural product biosynthetic pathways, and none pertain to light as a prerequisite for splicing. Our study addressed this knowledge gap and connected light and the regulation of a mushroom anabolic pathway via transcriptional regulation, combined with cotranscriptional alternative splicing, which now emerges as a regulatory principle in basidiomycete natural product metabolism.

## MATERIALS AND METHODS

### Microbial strains and growth conditions.

Escherichia coli XL1-Blue was cultivated in lysogeny broth (LB) medium (10 g tryptone, 5 g yeast extract, and 10 g NaCl [pH 7] per L) supplemented with 50 μg/mL carbenicillin at 37°C. *Terana caerulea* CBS 452.86 (wild type; Westerdijk Institute) was cultivated on malt extract-peptone (MEP) agar plates (30 g malt extract, 3 g Soytone peptone, and 18 g agar [pH 5.6] per L) for 14 days at 25°C in the dark and exposed to light, as indicated below. For stable-isotope labeling, 5 mM l-[3-^13^C]phenylalanine (CortecNet) or l-[3,5-D_2_]tyrosine (Cambridge Isotope Laboratories, Inc.) was added to the MEP agar. For genomic sequencing, *T. caerulea* was cultivated in liquid MEP medium at 25°C and shaken at 100 rpm for 10 days in the dark.

Aspergillus nidulans FGSC A4 (wild type; Fungal Genetics Stock Center) served as the host for heterologous protein production. A. nidulans strains were maintained on Aspergillus minimal medium (AMM) (60) agar plates, supplemented with 5 mM l-glutamine, at 37°C for 3 days. Plates for positive transformants were supplemented with 0.1 μg/mL pyrithiamine hydrobromide. Aspergillus conidia were harvested with 10 mL sterile phosphate-buffered saline (8 g NaCl, 0.2 g KCl, 1.44 g Na_2_HPO_4_, 0.24 g KH_2_PO_4_, and 0.5 mL Tween 20 [pH 7.2] per L), and the suspension was passed through a cell strainer (40 μm) (EASYstrainer). Media were inoculated at a titer of 1 × 10^6^ conidia per mL. To isolate genomic DNA (gDNA) to verify transgene integration, transformants were cultivated in yeast extract-peptone-dextrose (YPD) medium (20 g peptone, 20 g d-glucose monohydrate, and 10 g yeast extract [pH 6.5] per L) at 37°C for 24 h. A. nidulans strains generated in this study have the genotype P*alcA*-*corA* (cDNA)_*ptrA* (tStL04, to express *corA* cDNA) or P*alcA*-*corA* (gDNA)_*ptrA* (tStL06, to express *corA* gDNA).

To produce polyporic acid heterologously for preparative purposes, A. nidulans tStL04 was cultivated in 15 L autoinducing AMM (17), prepared with 200 mM ethanol and 5 mM d-glucose and 2 mM l-glutamine as the carbon and nitrogen sources, at 37°C at 180 rpm for 72 h.

### Nucleic acid isolation and first-strand synthesis.

For genomic sequencing, the gDNA was extracted using the cetyltrimethylammonium bromide (CTAB) protocol (61). Cells were resuspended in 500 μL TLB buffer (100 mM Tris-HCl, 70 mM EDTA, 2% [wt/vol] SDS, 2% [vol/vol] β-mercaptoethanol) and treated with 5 μL Monarch RNase A (20 mg/mL; New England BioLabs [NEB]) and then with 5 μL proteinase K (10 mg/mL; Merck), at 60°C for 1 h each. After the addition of 100 μL of 4 M NaCl and 100 μL of a CTAB solution (700 mM NaCl, 275 mM CTAB) and another incubation at 60°C for 1 h, the mixture was spun down, and the supernatant was extracted three times with phenol-chloroform-isoamyl alcohol (25:24:1, vol/vol/vol). The gDNA was precipitated with an equal volume of isopropanol and washed 10 times with ice-cold 70% ethanol. The genomic DNA pellet was dissolved in 10 mM Tris-HCl (pH 8.5).

To amplify intron-disrupted genes from *T. caerulea* gDNA or to verify transgene integration into A. nidulans, gDNA was extracted from the harvested mycelium from a liquid culture (see above), which was ground under liquid nitrogen. The homogenized mycelium was dissolved in LETS buffer (100 mM LiCl, 20 mM EDTA [pH 8], 10 mM Tris [pH 8], 0.5% [wt/vol] SDS) and treated with 1 μL Monarch RNase A (20 mg/mL) and then with 10 μL proteinase K (10 mg/mL), at 65°C for 20 min each. The mixture was spun down, and the supernatant was extracted three times with phenol-chloroform-isoamyl alcohol (25:24:1, vol/vol/vol). The gDNA was precipitated with an equal volume of ice-cold isopropanol with the addition of 40 μL 4 M NaCl. The gDNA pellet was washed twice with ice-cold 70% (vol/vol) ethanol, dried, and dissolved in nuclease-free water.

To isolate fungal RNA, the mycelium was ground under liquid nitrogen, and the total RNA was isolated using the SV total RNA isolation kit (Promega). Residual genomic DNA was removed with Baseline-Zero DNase (Biozym). For first-strand synthesis, 1 μg of the total RNA template was incubated with anchored (dT)_18_ oligonucleotides for 5 min at 65°C, and first-strand synthesis (20 μL final volume) was performed for 1 h at 42°C using a RevertAid reverse transcriptase kit (Thermo Fisher) according to the manufacturer’s instructions.

### Genome sequencing.

Genomic DNA sequencing of *T. caerulea* was carried out with 50 mg mycelia (10 days, in liquid MEP medium at 25°C with shaking at 100 rpm, in the dark) ground under liquid nitrogen. Genomic DNA was extracted as described above. To prepare a library for Nanopore sequencing, 500 ng DNA was processed with a rapid sequencing kit (Oxford Nanopore Technologies) according to the manufacturer’s instructions and sequenced on a MinION flow cell. The genome was assembled using CANU (62, 63) v.1.9., based on an expected genome size of 50 Mbp. Signal-level reads were indexed against the draft genome using Nanopolish software (64). After using minimap (65) and samtools (66) to sort and map the reads, a consensus sequence was calculated using Nanopolish (67). Gene models were individually verified with the Augustus algorithm in Aspergillus mode (68) and compared with BLAST (69).

### qRT-PCR.

For qRT-PCR, 4 μL of the first-strand reaction samples was diluted 1:4 in nuclease-free water prior to use. Thereafter, 4 μL was added to a reaction mixture composed of each gene-specific oligonucleotide (see Table S4 in the supplemental material) in quantitative PCR (qPCR) mix (EvaGreen; Bio&SELL) in a final volume of 20 μL. Oligonucleotides (200 nM) with a primer efficiency of at least 90% were used (Table S4). Reaction mixtures were run in technical triplicates in a qTOWER^3^G cycler (Analytik Jena). Thermal cycling conditions were an initial denaturation step at 95°C for 15 min; 40 cycles of 95°C for 15 s, 60°C for 20 s, and 72°C for 20 s (data acquisition); and a final melt analysis from 60°C to 95°C. qRT-PCR data were analyzed using qPCRsoft V4.0 software (Analytik Jena). Relative quantification was performed by using the 2^−ΔΔ^*^CT^* equation (70), using the annotated *T. caerulea ras* gene as the internal control, which showed constant expression throughout, which is consistent with the findings for other basidiomycetes (71). Experiments were run in at least biological triplicates from independent agar plates. *T. caerulea* cultures not exposed to light were used as a reference (relative quantity = 1). qRT-PCR controls included (i) template-free (nuclease-free water) reaction samples to check for interference and contamination and (ii) total RNA samples treated twice with DNase to check for gDNA contamination.

### Semiquantitative PCR.

The reaction mixtures contained MgCl_2_ (2 mM), deoxynucleoside triphosphates (dNTPs) (0.2 mM each), oligonucleotide primers (1 μM each) (Table S4), genomic DNA or the first-strand reaction sample as the template, and 0.5 U DreamTaq DNA polymerase (Thermo Fisher), with a total volume of 20 μL. Thermocycling conditions were an initial denaturation step for 2 min at 95°C, amplification for 35 cycles (95°C for 30 s, 60°C for 30 s, and 72°C for 15 s), and a terminal hold for 5 min at 72°C. PCR products were analyzed on a 2% (wt/vol) agarose gel, ligated to vector pJET (Thermo Fisher), and sequenced.

### Construction of the *corA* expression plasmids.

The *corA* coding sequence was amplified from the first-strand reaction mixture (see above). The reaction mixture contained MgCl_2_ (1.5 mM), dNTPs (0.2 mM each), oligonucleotide primers oSS138 and oSS127 (0.5 μM each) (Table S4), and 0.5 U Phusion high-fidelity DNA polymerase (NEB), in a total volume of 20 μL. Thermocycling conditions were an initial denaturation step for 2 min at 98°C, amplification for 35 cycles (98°C for 20 s, 60°C for 20 s, and 72°C for 2 min), and a terminal hold for 5 min at 72°C. In parallel, the intron-containing chromosomal *corA* sequence was PCR amplified from gDNA using the same parameters. The gel-purified fragments were ligated to pUC19, amplified from plasmid pMG49 (17), using oligonucleotides oMG468 and oMG469 (Table S4). The PCR product was treated with DpnI (NEB) to remove the methylated DNA template and subsequently used to construct expression plasmids pSS06 (*corA* cDNA) and pSS09 (chromosomal *corA*). Inserts and the vector backbone were ligated by the Gibson assembly strategy using the NEBuilder HiFi DNA assembly cloning kit (NEB). Plasmids pSS06 and pSS09 were used to transform E. coli XL1-Blue for plasmid propagation. Correct assembly was confirmed by DNA sequencing.

### Fungal transformation and transformant analysis.

To generate expression platform strains, wild-type A. nidulans FGSC A4 was selected as the parental strain, which was transformed with P*alcA*:*corA_ptrA* plasmids that contain the A. nidulans promoter P*alcA*, the *corA* gene (pSS06, cDNA of *corA*; pSS09, chromosomal *corA*), and the *ptrA* gene as a pyrithiamine resistance marker for the selection of transformants. The mycelium to generate protoplasts was inoculated with 2 × 10^6^ to 3 × 10^6^ conidia per mL in YPD medium and grown for 24 h at 37°C. A. nidulans protoplasts were obtained by the incubation of the mycelium with 1.1 g per 20 mL VinoTaste Pro (Novozymes) and 0.1 g per 20 mL Trichoderma harzianum lysing enzymes (Sigma) for 3 h in YAT buffer (0.6 M KCl, 50 mM maleic acid [pH 5.5]). Protoplasts were washed with solution A (0.6 M KCl, 50 mM CaCl_2_, 10 mM Tris-HCl [pH 7.5]), mixed with 10 μg of the respective plasmids and a polyethylene glycol (PEG) solution (25% PEG 8000, 50 mM CaCl_2_, 10 mM Tris-HCl [pH 7.5]), and incubated for 30 min on ice. After transformation, protoplasts were regenerated on AMM agar plates containing 1.2 M sorbitol for osmotic stability, 100 mM d-glucose, and 20 mM l-glutamine. Positive transformants were selected by resistance to pyrithiamine at a final concentration of 0.1 μg/mL (72).

To verify the genomic integration of *corA* into A. nidulans tStL04 and tStL06, PCR was performed with gDNA as the template, Phusion high-fidelity DNA polymerase (NEB) as described above for *corA* amplification, and oligonucleotides oMG234 and oMG169 (Table S4). For further work, one transformant was chosen for each plasmid (A. nidulans tStL04 for pSS06 and A. nidulans tStL06 for pSS09).

### Chromatographic analysis of natural products.

To detect corticins in the *T. caerulea* biomass, the mycelium was harvested from MEP plates, ground under liquid nitrogen, and extracted with ethyl acetate. The extract was evaporated to dryness under reduced pressure, dissolved in methanol (MeOH), centrifuged, filtered, and subjected to UHPLC-MS, carried out on an Agilent 1290 Infinity II chromatograph with a diode array detector coupled to a 6130 single quadrupole mass detector, run in electrospray ionization mode, using method I. For method I, the chromatograph was equipped with an Agilent Eclipse XDB C_18_ column (250 by 9.4 mm, 5-μm particle size, thermostatted at 30°C) and run with 0.1% formic acid in water (solvent A) and acetonitrile (ACN) (solvent B) at a flow rate of 1 mL/min. A linear gradient was applied, with an increase from 5 to 60% solvent B within 2 min, a further increase to 85% solvent B within 5 min, and, finally, an increase to 100% solvent B within 1 min.

To detect polyporic acid in culture filtrates of A. nidulans tStL04 and tStL06, the broth was acidified to pH 3 and extracted twice with ethyl acetate. Crude extracts were dried with Na_2_SO_4_, evaporated to dryness, and dissolved in MeOH. The extract was centrifuged, filtered, and subjected to UHPLC-MS analysis on the same instrument but using a Phenomenex Luna Omega Polar C_18_ column (50 by 2.1 mm, 1.6 μm, 30°C). A linear gradient with the same solvents A and B (flow rate of 0.85 mL/min) was applied, with an increase from 14 to 50% solvent B within 3 min and an increase to 100% solvent B within 30 s (method II). Data analysis was performed using ChemStation software (Agilent). High-resolution mass spectra and MS/MS fragmentation patterns were recorded using a Q Exactive Plus mass spectrometer (Thermo Scientific). Data analysis was performed using Xcalibur software (Thermo Fisher). The stable-isotope incorporation rate was calculated based on the absolute intensity of ions detected in the mass spectrum by the following equation (73):

$$\%\text{ enrichment }=\left(\frac{{\left[M+n\right]}_{\text{labeled}}}{{\left[M\right]}_{\text{labeled}}+{\left[M+n\right]}_{\text{labeled}}}\;-\;\frac{{\left[M+n\right]}_{\text{unlabeled}}}{{\left[M\right]}_{\text{unlabeled}}+{\left[M+n\right]}_{\text{unlabeled}}}\right)\times 100$$

where *M* is the unlabeled ion mass spectrum peak, *M* + *n* is the peak expected from the labeled ion, and “labeled” or “unlabeled” indicates cultures that were supplemented with either stable-isotope-labeled or unlabeled compounds, respectively.

### Isolation and analysis of polyporic acid.

The broth of a 15-L culture of A. nidulans tStL04 was separated from mycelia by filtering through miracloth, acidified to pH 3, and exhaustively extracted with ethyl acetate. The organic phase was dried with Na_2_SO_4_ and evaporated to dryness. The residue was dissolved in MeOH and subjected to size exclusion chromatography on a Sephadex LH-20 column (column dimensions of 50 by 3.2 cm), with MeOH as the eluent. Fractions containing polyporic acid were pooled, used for semipreparative high-performance liquid chromatography (HPLC) on an Agilent 1260 chromatograph equipped with an Agilent Eclipse XDB C_18_ column (250 by 9.4 mm, 5 μm), and run with 0.1% trifluoroacetic acid in water (solvent A) and ACN (solvent B) at a flow rate of 2.5 mL/min and a column temperature of 12°C. A linear gradient was applied, with an increase from 35 to 80% solvent B within 11 min and an increase to 100% solvent B within a further 6.5 min (method III). The isolated fractions were pooled, the organic solvent was removed under reduced pressure, and the extract was lyophilized. Compound 8 was dissolved in MeOH, chromatographically analyzed by UHPLC-MS using method II, and subsequently used for high-resolution mass spectrometry, tandem MS, and NMR spectroscopy. NMR spectra were recorded on a Bruker Avance III 600-MHz spectrometer at 300 K. Samples were solved in deuterated dimethyl sulfoxide (DMSO-*d*_6_). ^1^H and ^13^C NMR chemical shifts were referenced to solvent signals (δ_H_ = 2.50 ppm; δ_C_ = 39.5 ppm).

### Light exposure experiments.

*T. caerulea* grown on MEP agar plates was precultured in the dark for 14 days and subsequently exposed to either white or monochromatic light.

White-light exposure followed a cycle of approximately 12 h of light/12 h of darkness for the indicated time intervals (minimum of 1 h and maximum of 72 h). Exposure to monochromatic light was carried out using a light-emitting diode light source (StarLight; Opto-Electronics). The wavelength was 367 nm (spectrum half-width of 9 nm), 465 nm (spectrum half-width of 10 nm), 525 nm, 590 nm, or 625 nm (spectrum half-width of 5 nm for these sources). The mycelium was exposed to monochromatic light for 8 h per day, followed by 16 h of darkness, for a total of 72 h. For UV light, exposure was done for 1 h per day, followed by 23 h of darkness, for 72 h. Mycelium samples were taken at the indicated time points and immediately frozen in liquid nitrogen until further processing.

### Data availability.

Genomic sequence data (GenBank accession number JAKOGH000000000) and the sequence of the *corA* gene (GenBank accession number OM515349) have been deposited at GenBank and are publicly available.
