# Supplementary material for: Blue Light-Dependent Pre-mRNA Splicing Controls Pigment Biosynthesis in the Mushroom Terana caerulea
Source: Microbiol Spectr. 2022 Sep 12;10(5):e01065-22. doi: 10.1128/spectrum.01065-22 (PMC9603100; doi:10.1128/spectrum.01065-22)
Supplement: Supplemental file 1 — Download spectrum.01065-22-s0001.pdf, PDF file, 2.1 MB [file spectrum.01065-22-s0001.pdf]

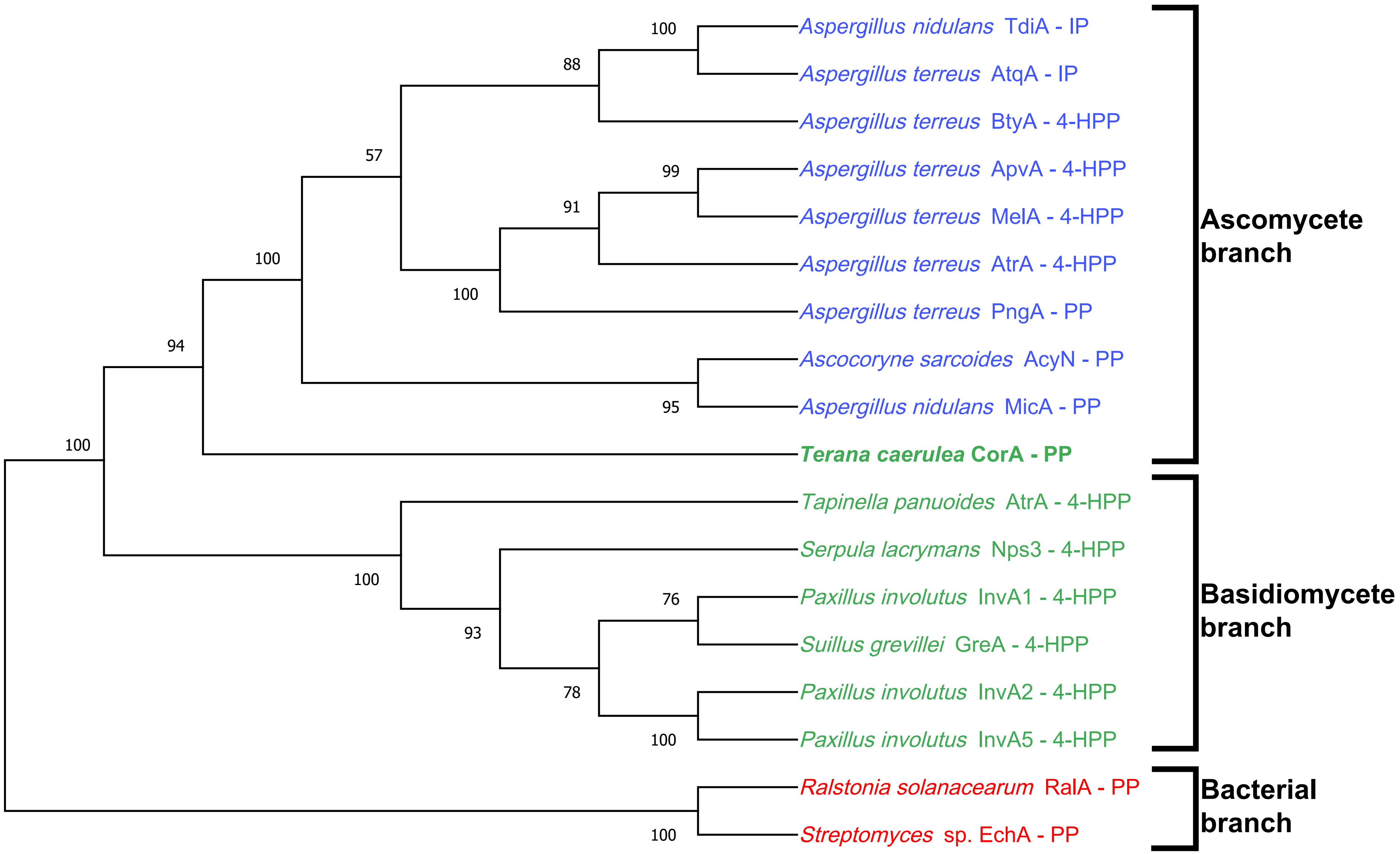

FIG S1. Phylogeny of CorA-related tri-domain synthetases, based on the alignment of their adenylation domains. Proteins used in this analysis are listed below. Enzymes are color-coded according to the taxonomic group in which they occur. Bacterial enzymes serve as outgroup. Substrates of the individual enzymes are indicated and abbreviated as follows: IP, indole-3-pyruvate; PP, phenylpyruvate; 4-HPP, 4-hydroxyphenylpyruvate.

This evolutionary history was inferred using the Maximum Likelihood method and Le_Gascuel_2008 model (1). The bootstrap consensus tree inferred from 1000 replicates (2) is taken to represent the evolution of the enzymes analyzed (2). Those branches are collapsed that correspond to partitions reproduced in less than 50% of bootstrap replicates. The percentage of replicate trees in which the associated taxa clustered together in the bootstrap test (1000 replicates) are shown next to the branches (2). Initial tree(s) for the heuristic search were obtained automatically by applying Neighbor-Join and BioNJ algorithms to a matrix of pairwise distances estimated using the JTT model (3), and then selecting the topology with superior log likelihood value. A discrete Gamma distribution was used to model evolutionary rate differences among sites (+G, parameter = 1.4248). The rate variation model allowed for some sites to be evolutionarily invariable ([+I], 5.27% sites). This analysis involved 18 amino acid sequences. All positions with less than 90% site coverage were eliminated, i.e., fewer than 10% alignment gaps, missing data, and ambiguous amino acids were allowed at any position (partial deletion option). There were a total of 484 positions in the final dataset. Evolutionary analyses were conducted in MEGA X (3).

Protein sequences used for the phylogenetic analysis of CorA and related enzymes.
Substrates are: IP, indole-3-pyruvate; PP, phenylpyruvate; 4-HPP, 4-hydroxyphenylpyruvate.

Organism	Enzyme	Substrate	Direct or pathway product	Uniprot/GenBank accession
**Basidiomycota**				
Terana caerulea	CorA	PP	Polyporic acid	OM515349
Tapinella panuoides	AtrA	4-HPP	Atromentin	B7STY1
Serpula lacrymans	NPS3	4-HPP	Atromentin	F8P1W3
Paxillus involutus	InvA1	4-HPP	Atromentin	A0A0S2E7Z1
	InvA2	4-HPP	Atromentin	A0A0S1RUN4
	InvA5	4-HPP	Atromentin	A0A0S2E7W7
Suillus grevillei	GreA	4-HPP	Atromentin	I6NXV7
**Ascomycota**				
Ascocoryne sarcoides	AcyN	PP	Polyporic acid	OL770279
Aspergillus terreus	ApvA	4-HPP	Aspulvinone E	Q0CWD0
Aspergillus terreus	MelA	4-HPP	Aspulvinone E	A0A336U965
Aspergillus terreus	AtrA	4-HPP	Atromentin	Q0CT94
Aspergillus terreus	PngA	PP	Phenguignardate	Q0CBN5
Aspergillus terreus	AtqA	IP	Didemethylasterriquinone D	Q0D034
Aspergillus nidulans	TdiA	IP	Didemethylasterriquinone D	A7XRY0
Aspergillus terreus	BtyA	4-HPP	Butyrolactone II	Q0CU19
Aspergillus nidulans	MicA	PP	Microperfuranone	Q5B7T4
**Bacteria**				
Streptomyces spec.	EchA	PP	Polyporic acid/ Echoside	AHN91924
Ralstonia solanacearum	RalA	PP	Ralfuranone I	HQ864831

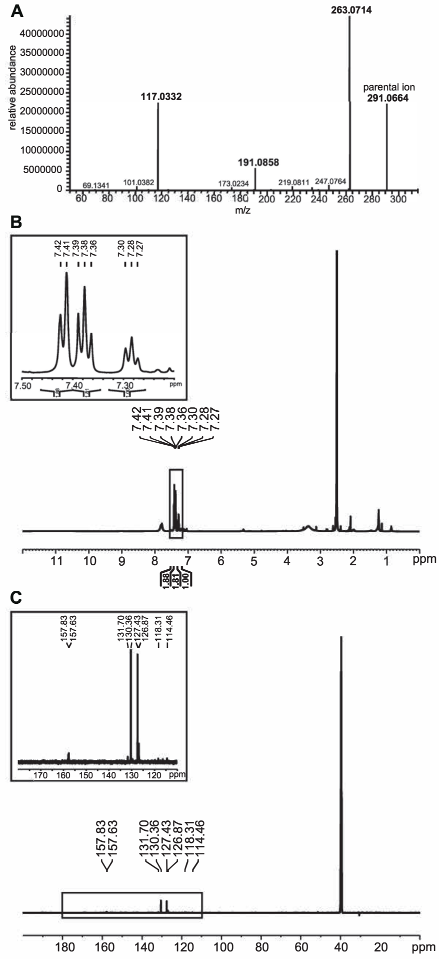

FIG S2. Analytical data of polyporic acid, isolated from *A. nidulans* tStL04.
A) MS/MS spectrum with characteristic fragmentation pattern
B) 1H NMR spectrum and
C) 13C NMR spectrum of polyporic acid.
The insets show the magnification of signals within the box.

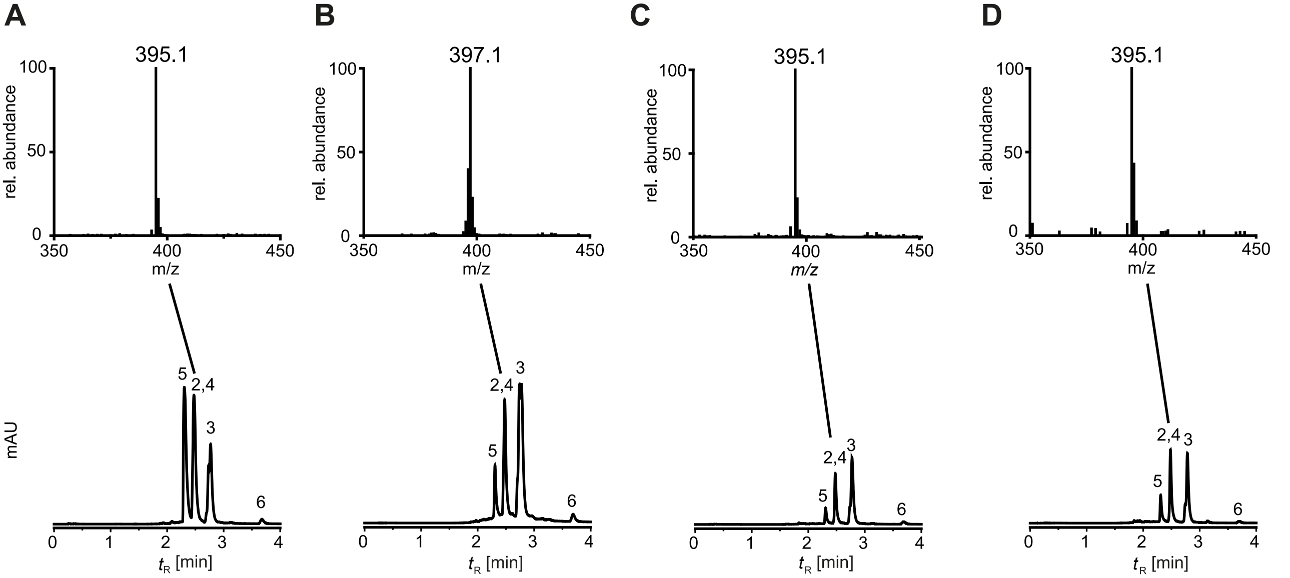

FIG S3. Analysis of corticins isolated from *T. caerulea* and mass spectra of corticins A/D after stable isotope labeling. Corticins were extracted with ethyl acetate isolated from light-exposed *T. caerulea* agar cultures and chromatographically analysed by UHPLC/MS. A) Corticins, isolated from a control where media was supplemented with 5 mM L-phenylalanine. B) Corticins, isolated from a culture supplemented with 5 mM L-[3-^{13}C]phenylalanine. The detected mass shift of *m/z* +1 or +2 reflects incorporation of one or two ^{13}C stable isotope labeled phenylalanine precursors. C) Corticins isolated form a control culture supplemented with 5 mM L-tyrosine. D) Corticine, isolated from a culture supplemented with 5 mM L-[3,5-D_2]tyrosine. Signals were detected at $\lambda = 358$ nm. Retention times are t_R = 2.3 min corticin E (**5**), t_R = 2.5 min corticin A/D (**2** and **4**), t_R = 2.7 min corticin B (**3**), and t_R = 3.7 min hexamethylthelephoric acid (**6**).

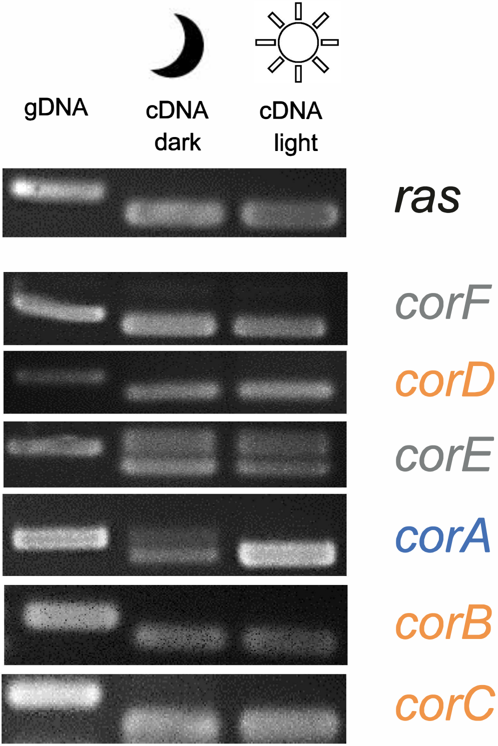

FIG S4. Semiquantitative reverse transcription PCR for *corA* and adjacent genes. As template, RNA was used that was either isolated from mycelium kept continuously in darkness or incubated for 72 h in a 12 h light/12 h dark cycle. For control, the constitutively expressed *ras* gene is shown in the upper panel. For comparison, a PCR product of the respective gene amplified from gDNA is shown in the left column.

A

1 gagagggcac gtggatacaa **ATG**AGTGTGC TGAGCGTGGT CGTCGAGGCC 50
51 GAGTTGAACG GCGGCCACCA CGTGCGGCGT GACAGCTCGC gtacctctgt 100
101 gacagacaga cgtgacagca tgtgacaatg cgtgtaagcc agGCTCGGGC 150
151 GTCTGGCGCG CCGCCGACGC CGCCGCTTTC CTGCGGTCAC TGCAGCCGGC 200
201 GATCTGCACG CCAACGCGTT GGGGCGTGCC GCATACGGCA AGGCACCgtc 250
251 attattaagc cattgacaaa catgtaatct cgttataatt ctgcagtaac 300
301 ctccgtgcca gACGCCGCTT CGCTGCAGGC TTCAGGCCAA ACGGAGCTGG 350
351 CGGGACTGTG CAGACGGCGG CAGCGGCAGG CGGCAAGGGG CGACGTTATG 400
401 GCAGCAGAGG CCCAGGGCGA GGGCGAGTAC GATTGGAACG GGGGAAAGgt 450
451 ccgtcccaaa acggcataat ccacaccggc ggcaagtcgg agccctcgag 500
501 gggccaaatt catcttgcgg aagcagACTC GGACAACATT ACTGCGTCAC 550
551 TCCTTCACCC ACCACCCGAC GACCTCACCC ACACCACCCC TCGCCGTCAT 600
601 CCACGACGAC GACGACGACG ACGACGGTGA CCACGAGCTC CGTCTTTTTC 650
651 ACGCTCACTC TCTTCCTTCA CCTCACCAGG CGCCTTGCAC CCCCGCCTCA 700
701 CCGCCTCCGC GTGCAGCCCC TGTCTGGACC GGGCACTCTT GTAACCGTGG 750
751 AAAATATCGC AGAGCTTCCT GCGCACGGCC CAAAATCTCG AGTTTTCGTC 800
801 AAATCTACTT TGAATCGCCG ATTGCCGCAG CTGCACTCTA TCTGCGTGCC 850
851 CACACCCAGG ACTTCCCCAC CAAGCGTTCA GGAAGGTCAA GACATTTCCA 900
901 AGTCGCCATC AGCCACCGCA AACAGTCTCA TAACGGCCCA CACAAgtaag 950
951 tgctcgtcca cggcctgtct ggtgggctct ttggctaacg tgtgctctat 1000
1001 ctgctctccg ccagatacca tgccattcga aagGTACTTA CAGTACAATG 1050
1051 CCGAACAAGA GCAGGCCAAC CAGCCGCGAG AGAGCCAGGA GCGCACTTCT 1100
1101 CTCTATGAAG ACTATTCGAA TCAGAGTCGC GCAGAGTCGT TCCAATACGA 1150
1151 TCCATCCATC TTCTCGGGCA ATGTCCAGTT CCAGATGCCT CAGCTTATGT 1200
1201 TAGGCCCCAC CGTCGCGCCC GGAGGAGGTG CGGAGGTTCT ACCAGCCGGT 1250
1251 ATCGTCGGGC GGTCAGCAGC AGTAGCTTCA AACTGGTTGG GGAATCCATC 1300
1301 GTACCGCAAC GCAAGCGAAG ACTAgtaggt gctatattcc cccgctgcta 1350
1351 gctgagttct catgcagtgc ttagCTCGAG TTGGTTCTTA TCGCCATCCA 1400
1401 TTCCTTCTTT CCCCTCACAG CCCATGGCAT CTCCTCCTAC CCATTTCCCG 1450
1451 ACTTCGCAGC AAGCTTCCTC AAGCGAGCCA GGTTCGCAGT CTCATTCGCA 1500
1501 ACGACCGTCG CATTCGCCTG TCGAATTCTC TACAGTTGCT TATAACTCGG 1550
1551 TTCACCCGCT TCAGGACGCT CTCGCTCAAC gtacaccaaa tattccctcc 1600
1601 tcgcgcgctg taccaatatc actgcctttc atcttacctt cttcagGGGG 1650
1651 CTTGCCGGTA TACTCTGCCT CTGGTTTCGA CATCCTGTCT ATCTTGGCTC 1700
1701 GCGTGGCTAC TCGGCCACAC CCGAAGATCG CGTTGGGCCC AGTAGACATG 1750
1751 ACCTGCTCAT TCGTAGTCTC CGACGTCCGA CGCTTTGATC ACCCCATCGT 1800
1801 ATATGCATCG CCAACGTTTT TGAAGCTTAC CGGCTACTCG GAGGAAGAGG 1850
1851 TCATTGGCCG CAACTGCCGC TTCTTGCAAG CGCCAGGAGG GCAGCTGCAG 1900
1901 AAGGGCGAGC CCCGTAGGCA CACCGCGCCG GACGCAGTGG CGCACATGCG 1950
1951 CAAGTCCCTG GTTGCCGACA AGGAGGTTCA AGTCAGTCTG GTGAACTATC 2000
2001 GGAAGAATGG ATCTGCGTTC ATCAACCTGG TCACGGTGAT CCCTATTCCT 2050
2051 GGCGGTGTCA GCAACCGACC AGAGGAAGCC GACGACATCG TGTACCAAGT 2100
2101 TGGCTTCCAG GTTGATTTGA CAGAGCAACC CAATGCTATT CTCCAAAGGG 2150
2151 TGCGAGACGG GAGCTACATG GCCAATTACA GCAACAATAT GGCATATCCC 2200
2201 TCTACGGCGG CGTCAAAGGA CTGGAAGATG AACTCTTCGA CGGCGGGCGT 2250
2251 CTCGAAGCAG TTCCGTAACG CTTTGGCCAA CCAAGATTTC TTGAGCAGCC 2300
2301 TTCCTATCAG CACCTCGACG ACGACGCTAT CGCTATCGGT GCAAGAGCGT 2350
2351 AACGACCCGT ACGACGGCAA CCGACCATTG AGCCTAATGT TGCTCGAGAC 2400
2401 CTCACCAGAC TTCGTCCATG TTCTTTCCTT GAAGGGCGCA TTCTTgtacg 2450
2451 tctctccctc ggtgaagcgg acattaggct acgaccctga ggagctggtc 2500
2501 ggcaagggga taaccgacta ctgccatgag GGCGGACAAG GTCCCCCTCG 2550
2551 TTCGCGAGCT CAAGGAGTCC TCCAGTATGG GCACTGGCGG TCCCAACGGC 2600
2601 TCAACGAAGG AGGAACCTGA GAGTCCGATA CAGCCCACGG CTCTGTCTCT 2650
2651 GGCGGCCGGC CCACGGGGCG TGGATCTCCT CTTCCGGATG CAGGCCAAGA 2700
2701 ACGGCGACTT CATCTGGGTC GAATGCAGCG GCCGGCTGTT TTTAGAGCCA 2750
2751 GGCAAGGGGC GCAAGGCCAT CATCCTCAGC GGTCGTGTGC GTGACATCCC 2800
2801 GCGCCTGCGG TGGCAGAGCG TTGCGCGCGC GGGCGGCCTG GCGCAGCCGG 2850
2851 ACACACAAAG CTCATCTTCG CGCGCGACGC CGCAAGGTTC AGAGGAAGCG 2900
2901 GCGGACCAGG AGCGGGAATG CTGGGCGCTG TTGAGTCTCG GCGGCTCGTT 2950
2951 CTTGCACGCG GGGACCGCGA TACGGGACCT GCTGGGGTGG AGCGTCGTCG 3000
3001 AGGTCATCGG CCGGTGTCTG AGCGACTTCA TCGGCGGGCG AGATCCGAGT 3050
3051 CAGGGCCGCG CGATGGTACA GGAGGCGCTG CAGCAGGCGT TCACTGACCC 3100
3101 GTCGTCGGAG AGCCGGAACC TGTCATGCGA ACTGCGGAAG AAAGACGGGA 3150
3151 CAGAGGTGAC GGTCGACGTC GTCCTGTACC ACCCAGAAGG GGATGCTGCC 3200
3201 CCTGCCATCG CGTCTGCGGC CACGACTCAG ATGCGTCCGC TCGTCTGCCA 3250
3251 GATCAAGCTC GCCGACGTGC CGCAGCTCGC AAACGCGGCC CTGATGGTCC 3300
3301 ACCCACACGG CGAAGACGTT TTCCTCGAGC TCGGCACGAG CCGGGGGAGC 3350
3351 GGGTGGCAGT ACGAGCTGCA GCAACTGAAG TACGCCAACC AGCGTCTGCT 3400
3401 GGAGGAGGTG GAGGAGCTCG AGTCGCGGGT GGAAGAGGAG CGGCAGGCGC 3450
3451 AGACTCAGGC CCAAGCGACG CAGCAGCGCG ACTTTGGTCG TGTCCGCCCG 3500
3501 TCGTACAGCA GCTCGTGGGC GAACCATCTC ATGAGTCATC CGGTACCTAC 3550
3551 CTCGCTGAAG CGTACATGGG ACGGACACGT CGTTGGGAGC AACGGAGACC 3600
4601 ACAGC**TAA**ct tatcatgcag tcgtcgta 3628

B

1 cactcacctc gtcggccacc **ATG**GAGGCTC AGCAGTCCAT CCCCCTCATT 50
51 CCTGCCCTCC CACCCGCCGG AACCTCCAGC TCTTCCAGAC AAGTGAACCA 100
101 GCAGCTCATA TTCGACTTTA CGAAGCGCAA GAGATGGGCC GACTTACTGG 150
151 TCACAGAGTT GACGGAAGTC GTCATGCTGG TGCTCTCTGA TACCGGCACC 200
201 GTCTGGTACT GCGGCCCAGC GGTGGAGGAT TTACTTGGTT GGGCGGATGA 250
251 GGAGCTTGTA GACGGCAACT TCTGCGAGAT CATGAATGgt gtgtcgcatc 300
301 cttgtgcgag ggcagcctca tcctcacccc tgagcagACC AAGACCGGAC 350
351 GACATTCGAG CGGCAGTTCC AAGAGTCTAT ACAGAATCGT ACCGACCTCG 400
401 TGGCCTATGC AAGACTACGC TGCAAGTCAG AGAACGACAT CTACCCTAAC 450
451 ATCCCTATCA TACCGACATT GCCCAATGTC GACGGCACGC CGCCACAAAC 500
501 TACGAGACCT CCGTCAAAGG AAATACTTTT CGAAATCAAA GGTTATCCGC 550
551 ACTATGTCAT CAGCTCGACT CTGACCAACC CGTCAAGCAA ATTCGATATC 600
601 CGGGACCTGA TGGGGAACAT GGGAGCTCAG GCAGGACCAT CATCCAGTTC 650
651 AGGTCAGGGA AGCGGGATAT TCAAGTGTTT CTTTGCCATG GCGAAACCAT 700
701 ACCCCAGCCG GAACACAGCC ATgtaagcga gtagacctga ttcacgcatc 750
751 cagtccgact gactcgtact tacagGCTGA ATACATTCCT GGAGCTCAAA 800
801 ATGGAGAACG AGAGGCTCCA GCAGCGTCTT CGCGATGCCA AATCGAACTC 850
851 CGAAGCCCTT GAGCTTGCCA AGGCCCAACG CCAGCAGTCG TCATATTCGT 900
901 ACGCTTACCA CTCACCCTAC GCCGCAACTT CGCATGGTCG GGCACCTACT 950
951 GGTCCACCGC CATCTCAATC CTTTGCTTGG ACGCTGAACT CGGCCGTCGC 1000
1001 TGGGCCTAGT CGCACGTTCG ACACCAGGCA GTTCTATCCA GGCTACCCCG 1050
1051 AGATCGCCAC CATGCCCGCC TCGAGCAGCT CCCCTAGCGC GATCGCGTCT 1100
1101 CCAACAAATA CAACCGCCGC CAGTCCAACA TCGGCGACCA ACCAGATGGA 1150
1151 CCCGGCGGCG GACCACACGC TGCCGCGCAA GAAGAGCCGG AAGTCGTTCT 1200
1201 CGCAGGACAT GTACTGCTGC ATGACGTGCG GGCGGACGGA TTCGCCGGAG 1250
1251 TGGCGGAAGg tgcgtgcact cgctttgatg taccggtatg tccgtggctc 1300
1301 aacatgtcat gatagGGCCC GCAAGGTCCC AAGACCTTGT GCAACGCATG 1350
1351 CGGGCTGCGC TGGGCGAAGT CTGTCCGCAG GGATCCCAGC CTGGCTGACG 1400
1401 ACGATAGAAA TGAT**TGA**ggt tggacgactc cccgcgg 1437

FIG S5. DNA sequences of putative *Terana caerulea* wc-1 homologous gene *twc1* (**A**) and wc-2 homologous gene *twc2* (**B**).
Grey: up- and downstream sequence, black capitalized: coding sequence in exons, black lower-case: introns.
Start and stop codons in bold.

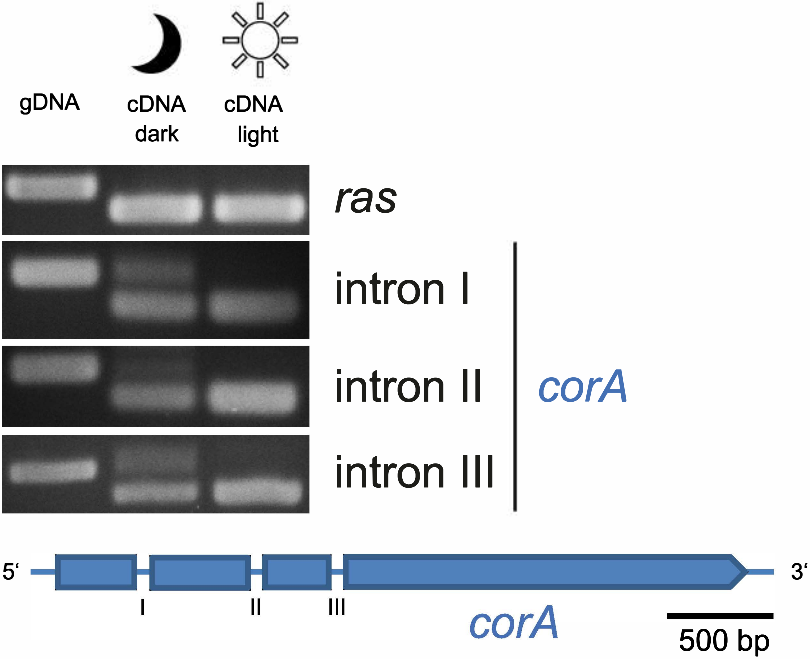

FIG S6. Semiquantitative reverse transcription PCR for intron I, II, and III of *corA*. As template, RNA was used that was either isolated from mycelium kept continuously in darkness or incubated for 72 h in a 12 h light/12 h dark cycle. For control, the constitutively expressed *ras* gene is shown in the upper panel. For comparison, a PCR product of the respective portion of the *corA* gene amplified from gDNA is shown in the left column. Amplicon sizes are:
312/255 bp (*ras*); 217/161 bp (amplicon across intron I);
210/146 bp (amplicon across intron II);
368/308 bp (amplicon across intron III of *corA*).

Table S1. HR-MS and MS^2 data of compounds **2** – **6** extracted from *T. caerulea*.

**Compound**	t_R **(min)**	**Formula**	**Neutral mass [*M*]**	**Found parental ion**	**HR-MS and MS^2 specific ions**			
					Ion mass	Formula	Origin	Reference
**2** and **4**	2.5	$C_{21}H_{16}O_8$	396.085	397.087			$[M+H]^+$	(4)
					382.063	$C_{20}H_{14}O_8$	$[M+H-CH_3]^+$	
					366.073	$C_{20}H_{14}O_7$	$[M+H-OCH_3]^+$	
					351.049	$C_{19}H_{11}O_7$	$[M+H-OCH_3-CH_3]^+$	
					335.018	$C_{18}H_7O_7$	$[M+H-OCH_3-(CH_3)_2]^+$	
**3**	2.7	$C_{22}H_{18}O_8$	410.100	411.103			$[M+H]^+$	(5)
					396.079	$C_{21}H_{16}O_8$	$[M+H-CH_3]^+$	
					380.088	$C_{21}H_{16}O_7$	$[M+H-OCH_3]^+$	
					365.065	$C_{20}H_{13}O_7$	$[M+H-CH_3-OCH_3]^+$	
					351.086	$C_{20}H_{15}O_6$	$[M+H-OCH_3-CO-H]^+$	
**5**	2.3	$C_{20}H_{14}O_8$	382.069	381.062			$[M-H]^-$	(4)
					366.038	$C_{19}H_{10}O_8$	$[M-H-CH_3]^-$	
					351.015	$C_{18}H_7O_8$	$[M-H-(CH_3)_2]^-$	
**6**	3.7	$C_{24}H_{22}O_8$	438.131	439.135			$[M+H]^+$	(6)
					424.111	$C_{23}H_{20}O_8$	$[M+H-CH_3]^+$	
					408.120	$C_{23}H_{20}O_7$	$[M+H-OCH_3]^+$	
					393.096	$C_{22}H_{17}O_7$	$[M+H-CH_3-OCH_3]^+$	
					378.076	$C_{21}H_{14}O_7$	$[M+H-(CH_3)_2-OCH_3]^+$	
					365.101	$C_{21}H_{17}O_6$	$[M+H-CO-OCH_3-CH_3]^+$	

Table S2. HR-MS, MS/MS, and NMR spectroscopic data of polyporic acid isolated from *A. nidulans* tStL04.[a] Experimentally determined NMR values are in agreement with literature data (7,8). Ph = phenyl.

Compound	t_R (min)	Formula	Neutral mass [*M*]	Parental ion (*m*/*z*)	Ion mass	Formula	Origin
Polyporic acid	2.8	$C_{18}H_{12}O_4$	292.0664	291.0664		$C_{18}H_{11}O_4$	$[M\text{-H}]^-$
					263.0714	$C_{17}H_{11}O_3$	$[M\text{-COH}]^-$
					191.0858	$C_{15}H_{11}$	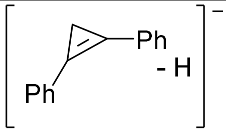
					117.0332	C_8H_5O	$[C_2OPh\text{-H}]^-$

Position	δ_C	δ_H, mult. (*J* in Hz), int.	HMBC
1	157.8	-	-
2	157.6	-	-
3	114.4	-	-
1´	131.7	-	-
2´	130.3	7.41, d (7.2), 4H	3, 2´,4´
3´	127.4	7.37, t (7.6), 4H	1´, 2´, 3´
4´	126.8	7.28, t (7.2), 2H	2´
2-OH	-	11.06, s (br)	-

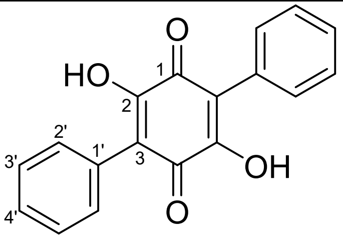

[a]DMSO-d_6, 600 MHz for ^{1}H and 150 MHz for ^{13}C NMR.

Table S3. Hypothetical or characterized WC-1 and WC-2-like proteins.

Protein	Organism	Length	Reference
WC-1	*Neurospora crassa*	1167 aa	(9)
Cwc1	*Cryptococcus neoformans*	1097 aa	(10)
PHRA	*Lentinula edodes*	924 aa	(11)
PoWC-1	*Pleurotus ostreatus*	651 aa	(12)
WC-1	*Schizophyllum commune*	843 aa	(13)
Twc1	*Terana caerulea*	1086 aa	this work
WC-2	*Neurospora crassa*	530 aa	(14)
Cwc2	*Cryptococcus neoformans*	392 aa	(10)
PHRB	*Lentinula edodes*	313 aa	(15)
PoWC-2	*Pleurotus ostreatus*	771 aa	(12)
WC-2	*Schizophyllum commune*	350 aa	(13)
Twc2	*Terana caerulea*	412 aa	this work

Table S4. DNA oligonucleotides used in this study.

**Cloning and sequencing**			
**Name**	**Gene**	**Sequence (5´- 3´)**	**Purpose**
oMG169	TtrpC	GCGCTTACACAGTACACGAGG	sequencing, genotyping
oMG234	PalcA	TTACGCCGGCGCGCCGTAGATATTTTCGAAGGGATTC	genotyping
oMG468	TrpcA	ACTTAACGTTACTGAAATCATCAAACAG	vector amplification
oMG469	PalcA	TTTGAGGCGAGGTGATAGGATTG	vector amplification
oMG474	PalcA	CATCCCCGCATAGCTGAACATC	sequencing
oSS07	*corA*	GGCCGTTCCCATCAAGACC	sequencing
oSS08	*corA*	CCTTCTCGTTGTTGTAGTACTCC	sequencing
oSS60	*corA*	CGTCTTCTACCAGCACAGTAGC	sequencing
oSS127	*corA*	GCTGTTTGATGATTTCAGTAACGTTAAGTTCACAGCGCAGCACTCGCG	cloning
oSS138	*corA*	CAATCCTATCACCTCGCCTCAAAATGCTATATAATCTCTCCTCGCCCGTC	cloning
**Semiquantitative PCR**			
oSS07	*corA*	GGCCGTTCCCATCAAGACC	
oSS08	*corA*	CCTTCTCGTTGTTGTAGTACTCC	
oSS11	*corC*	GCAAGAAGTTCAAGAACAACGG	
oSS12	*corC*	GTGCTCGGGCTTGGCGAA	
oSS17	*corE*	GAAGTGCACGCCGCAGTC	
oSS18	*corE*	GTCTGCGGCGTTGTCCTC	
oSS19	*corD*	TGGGAGCAAGAAGTTCAGGAA	
oSS20	*corD*	CGTTTGCACTCGTGTCGG	
oSS21	*corF*	CCGTCCTGTTCTCCAGCC	
oSS22	*corF*	CACTGCGAGATCTCTCCATCC	
oSS35	*ras*	CATGCGTACTGGAGAGGGC	
oSS36	*ras*	GCTTCGTCAACGTTGATGCG	
oSS101	*corB*	CGAAGACGCTCACGGAGATTG	
oSS102	*corB*	GCGTTGTTCTTGTAGGTGCG	
oSS128	*corA*	GGTACCGGCTGCACCATTC	
oSS129	*corA*	CCTCAACTGGATTGCCTTTGAC	
oSS130	*corA*	GGGACAGACGACAATCCCG	
oSS131	*corA*	CGTACAGGGAGCTCTACCTC	
**Quantitative RT-PCR**			**Efficiency**
oSS07	*corA*	GGCCGTTCCCATCAAGACC	0.96
oSS18	*corE*	GTCTGCGGCGTTGTCCTC	0.99
oSS19	*corD*	TGGGAGCAAGAAGTTCAGGAA	0.94 (0.9935)
oSS20	*corD*	CGTTTGCACTCGTGTCGG	0.94 (0.9935)
oSS21	*corF*	CCGTCCTGTTCTCCAGCC	0.95
oSS22	*corF*	CACTGCGAGATCTCTCCATCC	0.95
oSS64	*ras*	GCTGGACGTCTTGGATACCG	0.96
oSS65	*ras*	GCTGATTTCCTCGAACGAGTTTC	0.96
oSS72	corA	GGAGAGGTAGCGATCTGAGG	0.96
oSS75	*corC*	GTTGGAGCTCGGAGATCTTGG	1.01
oSS76	*corC*	CGTAGAGCCGAAGAAGTTGC	1.01

oSS80	*corE*	CCATCCACGGCATCCTGG	0.99
oSS101	*corB*	CGAAGACGCTCACGGAGATTG	0.91
oSS102	*corB*	GCGTTGTTCTTGTAGGTGCG	0.91
oSS140	*corA*	CTGTCCGGGTAAATGCGAC	0.90

References

1. Le SQ, Gascuel O. 2008. An Improved General Amino Acid Replacement Matrix. Mol Biol Evol 25:1307–1320.

2. Felsenstein J. 1985. Confidence limits on phylogenies: An approach using the bootstrap. Evolution 39:783–791.

3. Kumar S, Stecher G, Li M, Knyaz C, Tamura K. 2018. MEGA X: Molecular Evolutionary Genetics Analysis across computing platforms. Mol Biol Evol 35:1547–1549.

4. Maisterra M, Ángeles Castro M, Muñoz-Centeno LM, Calhelha RC, Ferreira IC, García PA. 2017. Cytotoxic Terphenyl Neolignans from Fungus *Terana coerulea*: New Natural Corticins D and E. and Revised Structure for Corticin A. Nat Prod Commun 12:695–698.

5. Briggs LH, Cambie RC, Dean IC, Hodges R, Ingram WB, Rutledge PS. 1976. Chemistry of fungi. XI. Corticins A, B, and C, benzobisbenzofurans from *Corticium caeruleum*. Aust J Chem 29:179–190.

6. Weisgraber K, Weiss U, Milne GWA, Silverton JV. 1972. Hexamethyl ether of leuco-thelephoric acid from *Corticium caeruleum*. Phytochemistry 11:2585–2587.

7. Jokela R, Lounasmaa M. 1997. p-Terphenyl- and phenanthraquinone derivatives: an NMR study. Planta Med. 63, 381–383. Planta Med 63:381–383.

8. Lohrisch HJ, Schmidt H, Steglich W. 1986. Fungal pigments. 50. Synthesis of terphenylquinones via methoxide-catalyzed rearrangement of grevillin derivatives. Liebigs Ann Chem 1986:195–204.

9. Ballario P, Vittorioso P, Magrelli A, Talora C, Cabibbo A, Macino G. 1996. White collar-1, a central regulator of blue light responses in *Neurospora*, is a zinc finger protein. EMBO J 15:1650–1657.

10. Idnurm A, Heitman J. 2005. Light controls growth and development via a conserved pathway in the fungal kingdom. PLoS Biol 3:e95.

11. Sano H, Narikiyo T, Kaneko S, Yamazaki T, Shishido K. 2007. Sequence Analysis and Expression of a Blue-Light Photoreceptor Gene, *Le.phrA* from the Basidiomycetous Mushroom *Lentinula edodes*. Biosci Biotechnol Biochem 71:2206–2213.

12. Qi Y, Sun X, Ma L, Wen Q, Qiu L, Shen J. 2020. Identification of two *Pleurotus ostreatus* blue light receptor genes (*PoWC-1* and *PoWC-2*) and *in vivo* confirmation of complex PoWC-12 formation through yeast two hybrid system. Fungal Biol 124:8–14.

13. Ohm RA, Aerts D, Wösten HAB, Lugones LG. 2013. The blue light receptor complex WC-1/2 of *Schizophyllum commune* is involved in mushroom formation and protection against phototoxicity. Environ Microbiol 15:943–955.

14. Linden H, Macino G. 1997. White collar 2, a partner in blue-light signal transduction, controlling expression of light–regulated genes in *Neurospora crassa*. EMBO J 16:98–109.

15. Sano H, Kaneko S, Sakamoto Y, Sato T, Shishido K. 2009. The basidiomycetous mushroom *Lentinula edodes* white collar-2 homolog PHRB, a partner of putative blue-light photoreceptor PHRA, binds to a specific site in the promoter region of the *L. edodes* tyrosinase gene. Fungal Genet Biol 46:333-341.
